# Cannabidiol Regulation of Learned Fear: Implications for Treating Anxiety-Related Disorders

**DOI:** 10.3389/fphar.2016.00454

**Published:** 2016-11-24

**Authors:** Regimantas Jurkus, Harriet L. L. Day, Francisco S. Guimarães, Jonathan L. C. Lee, Leandro J. Bertoglio, Carl W. Stevenson

**Affiliations:** ^1^School of Mathematical Sciences, University of NottinghamUniversity Park, Nottingham, UK; ^2^School of Biosciences, University of NottinghamLoughborough, UK; ^3^Department of Pharmacology, University of São PauloSão Paulo, Brazil; ^4^School of Psychology, University of BirminghamBirmingham, UK; ^5^Department of Pharmacology, Federal University of Santa CatarinaFlorianopolis, Brazil

**Keywords:** cannabidiol, extinction, fear conditioning, reconsolidation

## Abstract

Anxiety and trauma-related disorders are psychiatric diseases with a lifetime prevalence of up to 25%. Phobias and post-traumatic stress disorder (PTSD) are characterized by abnormal and persistent memories of fear-related contexts and cues. The effects of psychological treatments such as exposure therapy are often only temporary and medications can be ineffective and have adverse side effects. Growing evidence from human and animal studies indicates that cannabidiol, the main non-psychotomimetic phytocannabinoid present in *Cannabis sativa*, alleviates anxiety in paradigms assessing innate fear. More recently, the effects of cannabidiol on learned fear have been investigated in preclinical studies with translational relevance for phobias and PTSD. Here we review the findings from these studies, with an emphasis on cannabidiol regulation of contextual fear. The evidence indicates that cannabidiol reduces learned fear in different ways: (1) cannabidiol decreases fear expression acutely, (2) cannabidiol disrupts memory reconsolidation, leading to sustained fear attenuation upon memory retrieval, and (3) cannabidiol enhances extinction, the psychological process by which exposure therapy inhibits learned fear. We also present novel data on cannabidiol regulation of learned fear related to explicit cues, which indicates that auditory fear expression is also reduced acutely by cannabidiol. We conclude by outlining future directions for research to elucidate the neural circuit, psychological, cellular, and molecular mechanisms underlying the regulation of fear memory processing by cannabidiol. This line of investigation may lead to the development of cannabidiol as a novel therapeutic approach for treating anxiety and trauma-related disorders such as phobias and PTSD in the future.

## Introduction

Anxiety and trauma-related disorders will affect up to one in four people in their lifetime. Diseases like phobias and post-traumatic stress disorder (PTSD) show abnormal persistence of fear memories and can be debilitating, causing a huge societal and economic burden. Psychological treatments like exposure therapy are used to treat these disorders but they often show only limited or temporary effectiveness. Medications are also available but these are not fully effective in a significant proportion of patients, can have adverse side effects, and may even interfere with the efficacy of psychological therapy. There is therefore an urgent need for better treatments for these disorders (Fineberg et al., 2013; Baldwin et al., 2014).

**Figure 1:**
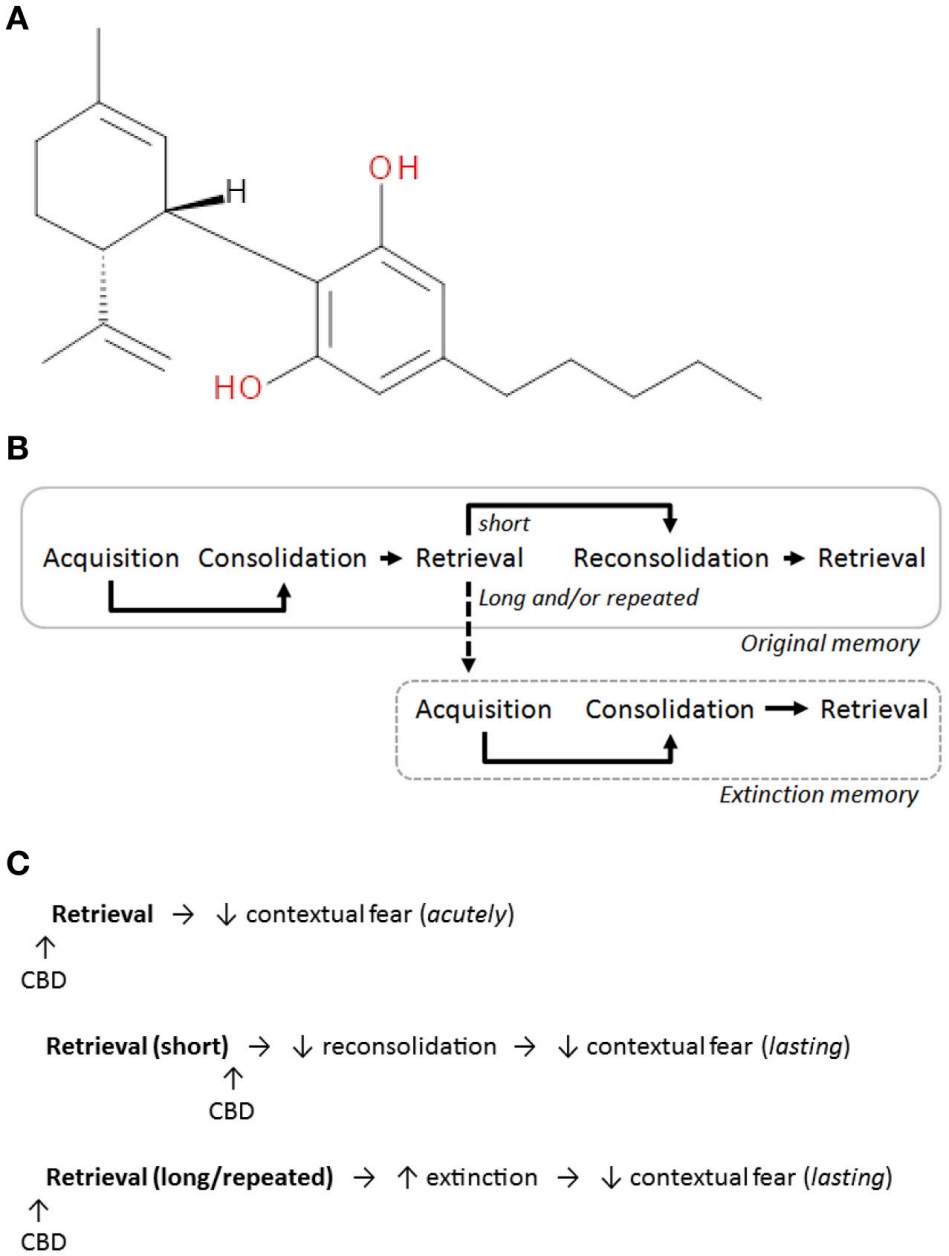
**(A)** The chemical structure of CBD (National Center for Biotechnology Information, 2016). **(B)** The different phases of fear memory. In the hours after its acquisition fear memory undergoes consolidation. After a short duration of retrieval, fear memory can become destabilized, after which it undergoes reconsolidation to maintain or update the memory. With longer retrieval and/or repeated exposures extinction can occur, resulting in the acquisition and consolidation of a new extinction memory which competes with the original memory to inhibit fear expression. **(C)** A summary of the effects of acute CBD administration on different contextual fear memory processes. CBD reduces learned fear expression, disrupts fear memory reconsolidation, and facilitates fear extinction.

A promising area of research in this field focuses on repurposing existing and developing novel drugs to enhance the effectiveness of psychological therapies in alleviating fear-related symptoms (Myers and Davis, 2007; Steckler and Risbrough, 2012). One drug showing broad therapeutic potential in various psychiatric diseases is cannabidiol (CBD), the main non-psychotropic constituent of the *Cannabis sativa* plant (Izzo et al., 2009; see chemical structure of CBD in Figure 1A). Studies in humans demonstrate the promise of CBD for treating anxiety (Blessing et al., 2015) and preclinical studies in rodents are elucidating the pharmacological mechanisms underlying its acute anxiolytic effects. These mechanisms include potentiation of serotonin (5-HT) transmission via 5-HT_1A_ receptor (5-HT_1A_R) activation and elevation of endocannabinoid levels via inhibition of their metabolism and re-uptake, which indirectly facilitates cannabinoid receptor type1 (CB1R) activation (for a review see Campos et al., 2016).

As well as reducing anxiety in behavioral tests of unconditioned fear, emerging evidence indicates that CBD regulates fear learning and memory in paradigms that are translationally relevant to diseases such as phobias and PTSD, along with their psychological treatment. In this paper we review the recent studies on CBD regulation of fear memory processing, which have focused on contextual fear. We also present novel data on CBD regulation of auditory fear memory and its extinction, which forms the theoretical basis for exposure therapy. We then outline future directions for research on this topic to gain a broader perspective on the neural circuit, psychological, pharmacological, and cellular bases of the regulation of learned fear by CBD.

## CBD regulation of contextual fear memory processing

Recent evidence indicates that CBD modulates fundamental neurobiological processes involved in Pavlovian fear conditioning, a form of associative learning by which certain stimuli or environments become predictive of threat and therefore enhance survival. During acquisition a neutral conditioned stimulus (CS) is associated with an aversive unconditioned stimulus (US), such as a mild footshock. The CS can be either discrete (i.e., cued), such as a light or tone, or the environment (i.e., context) where the US was presented. CS re-exposure after conditioning initially induces a fear response, which has frequently been inferred from behavioral (e.g., freezing) and/or autonomic (increased heart rate/blood pressure, decreased body temperature) changes (Fendt and Fanselow, 1999; Resstel et al., 2009). After its acquisition the CS-US association is consolidated into long-term fear memory. Later retrieval can render fear memory labile through destabilization of the memory trace, allowing for maintenance or updating of the memory through its reconsolidation (Lee, 2009). Extinction of fear memory occurs with longer durations or repeated sessions of retrieval. This form of inhibitory learning results in the encoding of a new CS-no US association which suppresses fear expression by competing with the original fear memory (Myers and Davis, 2007). Figure 1B depicts the different phases of fear memory and its possible reconsolidation or extinction after retrieval.

Accumulating evidence indicates that CBD regulates different contextual fear memory processes. An initial study by Resstel et al. (2006) showed that systemic CBD administration decreases the freezing response and autonomic changes induced by exposure to an aversively conditioned context; this effect was similar to the positive control diazepam. Subsequent studies confirmed the CBD-induced reduction in conditioned freezing expression with acute administration before retrieval (Lemos et al., 2010) or acquisition (Levin et al., 2012). In contrast, ElBatsh et al. (2012) showed that repeated daily injections (14 days) of CBD increased freezing expression during contextual fear retrieval. Chronic treatment with CBD has, however, been shown to facilitate adult hippocampal neurogenesis (Wolf et al., 2010; Campos et al., 2013), which is involved in aversive learning and memory processing as its facilitation enhances contextual discrimination and related fear expression (Efstathopoulos et al., 2015). Mice with reduced neurogenesis, on the other hand, presented less contextual fear (Pan et al., 2012; Denny et al., 2014). Both associative (through facilitation of associative learning) and non-associative (by buffering non-associative, anxiogenic effects of the aversive experience) mechanisms seem to play a role in regulating fear learning by adult hippocampal neurogenesis (Seo et al., 2015). Since the animals were conditioned during chronic CBD treatment, facilitated contextual fear learning due to enhanced hippocampal neurogenesis could explain the results reported by ElBatsh et al. (2012). This idea is supported by studies which showed that chronic CBD treatment rescues deficits in other types of memory in animal models of cognitive impairment (Fagherazzi et al., 2012; Cheng et al., 2014).

In addition to reducing contextual fear acutely when given before retrieval, CBD has also been shown to have enduring effects on fear expression when given in conjunction with memory reactivation (leading to reconsolidation) or extinction. Systemic CBD administration immediately after briefly retrieving a contextual fear memory disrupted its reconsolidation, resulting in a lasting reduction in fear expression during later retrieval (Stern et al., 2012, 2015; Gazarini et al., 2014). This effect depended on the (indirect) activation of CB1Rs rather than 5-HT_1A_Rs as prior antagonism of CB1Rs, but not 5-HT_1A_Rs, blocked the disruptive effect of CBD on reconsolidation (Stern et al., 2012). In contrast to its effect on the reconsolidation of contextual fear memory, CBD potentiated contextual fear extinction but this also results in reduced fear expression at later retrieval. Intracerebroventricular infusion of CBD before three longer retrieval sessions facilitated the extinction of contextual fear, an effect mediated indirectly via CB1R activation as it was blocked by CB1R antagonist pretreatment (Bitencourt et al., 2008). Systemic CBD administration before a single long retrieval session also affects contextual fear extinction but this depends on the strength of prior fear conditioning. Whereas, CBD enhanced the extinction of contextual fear resulting from strong conditioning, it impaired the contextual fear extinction induced by weaker conditioning (Song et al., this issue). Table 1 and Figure 1C summarize the reported effects of systemic CBD administration on contextual fear memory processing.

**Table 1:** **Summary of systemic CBD effects on contextual fear memory processing in male rats (Δ9-THC, Δ9-tetrahydrocannabinol; BDNF, brain derived neurotrophic factor; CB1R, cannabinoid type1 receptor; ERK1/2, extracellular signal-regulated kinase1/2; i.p., intraperitoneal; PL, prelimbic; SHR, spontaneously hypertensive rat; TrkB, tyrosine receptor kinase B)**.

**Strain**	**Dosing details**	**Effect**	**Possible mechanism(s)**	**References**
Wistar	10 mg/kg, i.p., pre-retrieval	Anxiolytic (↓ fear expression)	Not tested	Resstel et al., 2006
Lister hooded	10 mg/kg daily for 14 days, i.p., pre-acquisition and -retrieval	Anxiogenic (↑ fear expression) and/or potentiated fear conditioning	↓ hippocampal BDNF and TrkB expression, ↓ frontal cortex phospho-ERK1/2 levels	ElBatsh et al., 2012
Wistar and SHR	1.0–15 mg/kg, i.p., pre-acquisition	Anxiolytic (↓ fear expression) and/or disrupted fear memory formation (in Wistar rats only)	Not tested	Levin et al., 2012
Wistar	3.0–30 mg/kg, i.p., post-retrieval	Disrupted memory reconsolidation (bell-shaped dose-response curve)	Indirect CB1R activation	Stern et al., 2012
Wistar	10 mg/kg, i.p., post-retrieval	Disrupted memory reconsolidation	Not tested	Gazarini et al., 2014
Wistar	10 mg/kg, i.p., post-retrieval	Disrupted memory reconsolidation	Indirect CB1R activation in PL	Stern et al., 2014
Wistar	1.0 mg/kg + Δ9-THC 0.1 mg/kg, i.p., post-retrieval	Disrupted memory reconsolidation	Not tested	Stern et al., 2015
Lister hooded	10 mg/kg, i.p., pre-extinction (after weak or strong fear conditioning)	Impaired extinction with weak fear conditioning and enhanced extinction with strong fear conditioning	Not tested	Song et al., this issue

Recent studies have begun to determine the neural substrates mediating the effects of CBD on contextual fear memory processing. Lemos et al. (2010) initially investigated the brain sites in which CBD acts when attenuating the expression of learned fear. They reported that systemic CBD pretreatment prevented the increase in c-Fos expression induced by re-exposure to the conditioned context in the prelimbic (PL) and infralimbic (IL) subregions of the medial prefrontal cortex (mPFC) and the bed nucleus of the stria terminalis (BNST), brain areas which play key roles in fear regulation (Dejean et al., 2015; Lebow and Chen, 2016). Subsequently, the effect of direct CBD infusion into these brain regions on contextual fear memory retrieval was investigated. There was reduced conditioned freezing expression after its infusion into the PL or BNST (Lemos et al., 2010; Gomes et al., 2012; Fogaça et al., 2014). When infused into the IL, however, CBD produced the opposite effect (Lemos et al., 2010; Marinho et al., 2015). Convergent evidence suggests that the PL and IL present distinct neuroanatomical connections and functional roles (Hurley et al., 1991; Condé et al., 1995; Vertes, 2004), including the regulation of learned fear expression (Vidal-Gonzalez et al., 2006; Fenton et al., 2014). Contrasting effects of CBD after PL or IL infusion have also been reported in unlearned tests of anxiety (e.g., elevated plus maze), in which it induced anxiogenic- or anxiolytic-like effects, respectively (Fogaça et al., 2014; Marinho et al., 2015). Restraint stress pre-exposure reversed these effects, indicating that the stress experience could modulate the response of these mPFC subregions to aversive stimuli (Fogaça et al., 2014; Marinho et al., 2015). CBD regulation of contextual fear expression in these regions depends on 5-HT_1A_R activation as it is prevented by local 5-HT_1A_R antagonist pretreatment (Gomes et al., 2012; Fogaça et al., 2014; Marinho et al., 2015). CBD disruption of fear memory reconsolidation depended on CB1R but not 5-HT_1A_R activation in the PL (Stern et al., 2014). CBD infused into the IL cortex facilitated contextual fear extinction in a CB1R-dependent manner (Do Monte et al., 2013). Table 2 summarizes the reported effects of central CBD infusion on contextual fear memory processing.

**Table 2:** **Summary of central CBD effects on contextual fear memory processing in male rats (5-HT_1A_R, serotonin1A receptor; BNST, bed nucleus of the stria terminalis; CB1R, cannabinoid type1 receptor; i.c.v., intracerebroventricular; IL, infralimbic; i.p., intraperitoneal; PL, prelimbic)**.

**Strain**	**Dosing and site details**	**Effect**	**Possible mechanism**	**References**
Wistar	6.4 nmol, i.c.v., pre-retrieval	Facilitated fear extinction	Indirect CB1R activation	Bitencourt et al., 2008
Wistar	30 nmol, PL, pre-retrieval	Anxiolytic (↓ fear expression)	Not tested	Lemos et al., 2010
Wistar	30 nmol, IL, pre-retrieval	Anxiogenic (↑ fear expression)	Not tested	Lemos et al., 2010
Wistar	30–60 nmol, BNST, pre-retrieval	Anxiolytic (↓ fear expression)	5-HT_1A_R activation	Gomes et al., 2012
Long evans	1.3 nmol, IL, pre-extinction	Facilitated fear extinction	Indirect CB1R activation	Do Monte et al., 2013
Wistar	30 nmol, PL, pre-retrieval	Anxiolytic (↓ fear expression)	5-HT_1A_R activation	Fogaça et al., 2014
Wistar	30 nmol, IL, pre-retrieval	Anxiogenic (↑ fear expression)	5-HT_1A_R activation	Marinho et al., 2015

## CBD regulation of fear memory processing involving discrete cues

**Figure 2:**
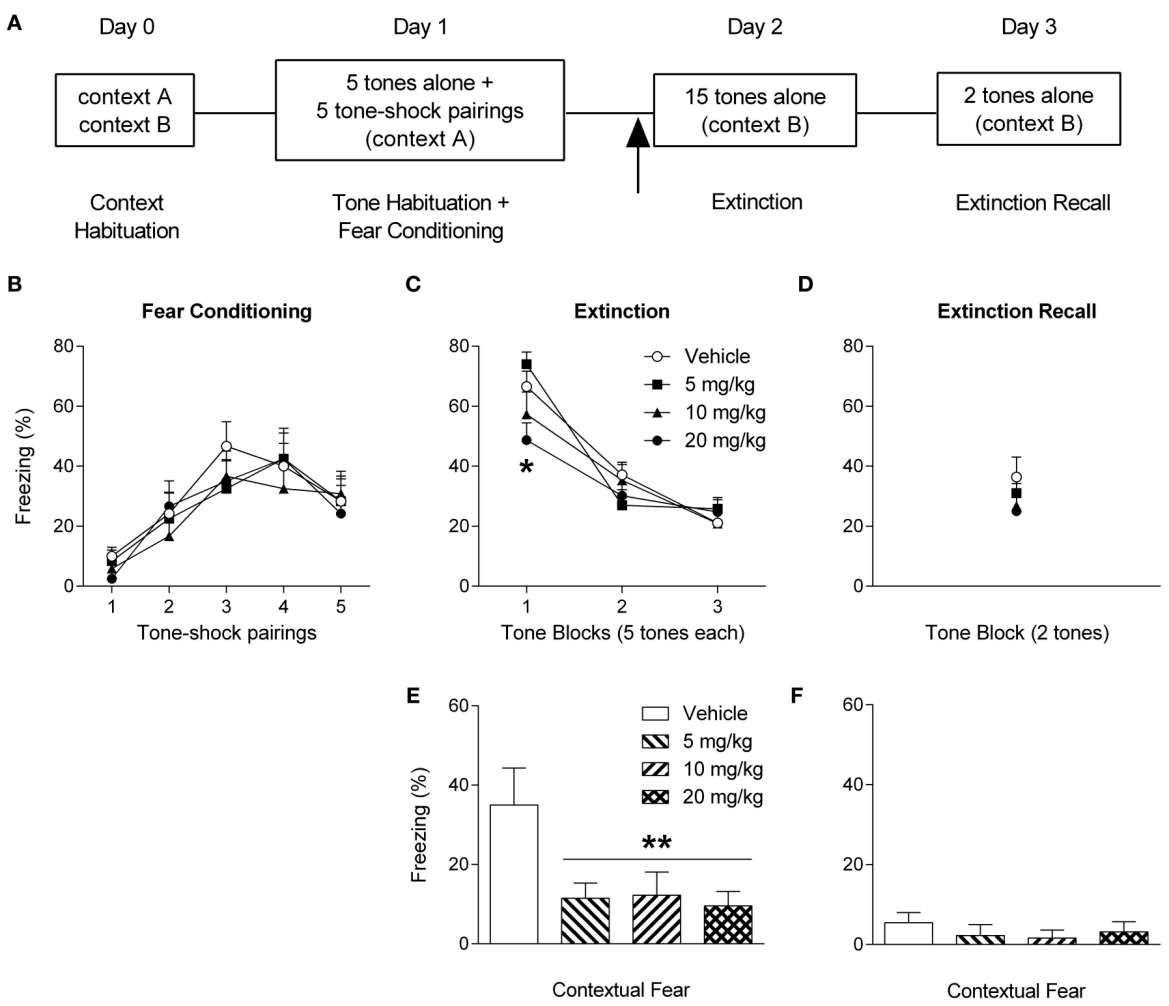
**Cannabidiol reduces the expression of auditory and contextual fear memory in rats. (A)** Schematic representation of the experimental procedures used; the arrow indicates that drug was injected 30 min before extinction. **(B)** Freezing during tone-shock pairings did not differ between the groups during auditory fear conditioning. **(C)** Rats treated with 20 mg/kg of cannabidiol showed significantly decreased freezing during tone presentations at the start of extinction, compared to rats given 5 mg/kg or vehicle (^*^P <0.05). **(D)** There were no differences in tone-induced freezing between the groups during extinction recall. **(E)** Rats treated with 5, 10, or 20 mg/kg of cannabidiol showed significantly decreased freezing in the 2 min period before tone presentations during extinction, compared to vehicle-treated controls (^**^P <0.01). **(F)** There were no differences in freezing between the groups in the 2 min period before tone presentations during extinction recall (see Supplementary Material for more details).

In contrast to contextual fear memory processing, few studies have examined the effects of CBD on learned fear related to discrete cues. One study in humans showed that CBD enhanced the extinction of visual fear memory when given immediately after, but not before, extinction (Das et al., 2013). More recently it was found in rats that CBD infused into the nucleus accumbens shell impaired the encoding of olfactory fear memory, an effect blocked by 5-HT_1A_R antagonism (Norris et al., 2016). To investigate this issue further we determined the effect of systemic CBD administration on the expression and extinction of auditory fear memory. Male Lister hooded rats were habituated to two distinct contexts (A and B), subjected to auditory fear conditioning (tone habituation and tone-shock pairings) in context A, treated with CBD (0, 5, 10, or 20 mg/kg, i.p.; *n* = 10–11/group) 30 min before undergoing extinction training (tone presentations) in context B, and underwent extinction recall testing (tone presentations) in context B (Figure 2A).

We found no differences in freezing during fear conditioning between the groups to receive different doses of CBD before extinction training (Figure 2B). Despite the relatively low levels of freezing during tone-shock pairings, conditioning resulted in robust fear memory encoding as indicated by the high freezing levels in response to tones during early extinction in vehicle-treated rats (Figure 2C). During extinction the high dose of CBD significantly decreased tone-induced freezing early in the session, compared to vehicle and the low dose. However, there were no differences in freezing during tone presentations later in the session or during extinction recall testing the next day (Figure 2D). This suggests that the high CBD dose decreased auditory fear memory expression without affecting its extinction. Although extinction occurred in a separate context to fear conditioning, vehicle-treated rats did show freezing in the period before tone presentations, indicating contextual fear expression at the start of the session (Figure 2E). We found that all three doses of CBD significantly decreased this contextual fear in comparison to vehicle. However, there were no differences in contextual fear expression before extinction recall testing (Figure 2F), suggesting that CBD acted acutely to reduce contextual fear during extinction.

Taken together, our results broadly confirm previous findings demonstrating that acute CBD treatment reduces contextual fear memory expression and extend them by showing that CBD also has a similar effect on the expression of auditory fear memory. Given that previous studies have shown a bell-shaped dose-response curve in relation to CBD regulation of anxiety and fear memory processing (Guimarães et al., 1990; Lemos et al., 2010; Stern et al., 2012), it is worth noting that we found that (1) the effect of CBD on contextual fear expression showed no dose-dependency, and (2) the dose of CBD needed to reduce auditory fear expression was much higher than for contextual fear. Moreover, CBD had no effect on auditory fear extinction. Although, this result agrees with the study of Das et al. (2013), which showed that CBD had no effect when given before cued fear extinction, it contrasts with the reported facilitatory effects of CBD on contextual fear extinction. The reasons for these discrepancies remain unclear but they could involve differences in the neural circuit and/or pharmacological mechanisms underlying CBD regulation of contextual vs. auditory fear memory expression (see below).

## Future directions

### Neural circuit and psychological mechanisms

As well as the mPFC, the neural circuitry underpinning fear memory processing comprises other inter-connected areas such as the hippocampus, amygdala, and periaqueductal gray (PAG) (Dejean et al., 2015). Infusing CBD into the PAG decreases anxiety in paradigms assessing innate fear (Campos and Guimarães, 2008). However, this area is also important for mediating freezing and other defensive behaviors in response to learned threats, suggesting that CBD regulation of fear memory expression may involve the PAG. In humans CBD reduces anxiety and autonomic arousal during the viewing of fearful facial expressions, which is accompanied by decreased activity in and functional connectivity between the amygdala and mPFC (Fusar-Poli et al., 2009, 2010). In mice CBD decreases amygdala activation as measured by c-Fos expression (Todd and Arnold, 2016). This raises the possibility that the amygdala plays a role in mediating CBD regulation of fear memory. CBD also reduces mPFC-hippocampus functional connectivity during cognitive processing (Bhattacharyya et al., 2015). This suggests that the hippocampus, which is crucial for contextual processing in particular, may also be involved in CBD regulation of certain aspects of learned fear. After extinction, fear expression is low when tested in the extinction context but fear renewal occurs outside of this context. This contextual regulation of fear extinction involves the hippocampus and its connections with the mPFC and amygdala (Maren et al., 2013). Although, we found no effects of CBD on auditory fear extinction, CBD might reduce fear renewal and/or the spontaneous recovery of fear that occurs over time after extinction through its actions on the hippocampus-mPFC-amygdala circuit. CBD modulation of this circuitry may also regulate learned fear in other paradigms with translational relevance. Phobias and PTSD are characterized by overgeneralization of fear to harmless discrete or contextual stimuli (Dunsmoor and Paz, 2015). A recent study showed that enhancing memory destabilization in combination with reconsolidation disruption by CBD reduced contextual fear generalization (Gazarini et al., 2014).

### Cellular and molecular mechanisms

As mentioned above, CBD regulates contextual fear memory processing in a 5HT_1A_R- and CB1R-dependent manner. It is unclear whether the action of CBD via 5-HT_1A_Rs to reduce fear memory expression is mediated by intracellular mechanisms. While 5-HT_1A_Rs certainly modulate intracellular kinases, the direction of modulation appears not to be consistent with fear inhibition. The 5-HT_1A_R activates protein kinase C (PKC) and extracellular signal-regulated kinase (ERK) (Raymond et al., 2001), but it is inhibition of PKC (Vianna et al., 2000) and ERK (Szapiro et al., 2000; Chen et al., 2005) that impairs fear memory expression. It should be noted, however, that these observations were made with intra-hippocampal drug infusions, whereas the acute effect of CBD has been shown to be mediated by the PL and BNST. Nevertheless, the putative action of CBD at 5-HT_1A_Rs might be mediated not via protein kinases, but by inhibition of adenylyl cyclase (AC). Such inhibition of AC and downstream dysregulation of second messenger systems might be expected ultimately to affect ongoing protein synthesis. Lopez et al. (2015) showed that inhibition of ongoing protein synthesis in the amygdala impaired fear memory expression, and so a similar perturbation of normal *de novo* protein synthesis by CBD via 5-HT_1A_Rs might be expected to impact upon retrieval. Speculatively, a pharmacologically-induced increase in protein synthesis might also sufficiently dysregulate the cellular mechanisms of retrieval to reduce fear expression. Therefore, any impact of CBD on protein synthesis could account for its acute effect on contextual, and cued, fear memory expression.

The actions of CBD to enhance extinction and impair reconsolidation both depend upon CB1Rs. Therefore, the coupling of CB1R activation to intracellular cascades may provide the cellular mechanism of long-term fear reduction. At the cellular level, mechanisms of reconsolidation tend to be conserved with those of extinction, such that inhibition results in impairment of both with opposite behavioral outcomes. A primary example of this is the effect of protein synthesis inhibitors, such as anisomycin, which impair reconsolidation to reduce fear expression as well as disrupting extinction to enhance subsequent fear expression. Therefore, in general terms, CB1R activation by CBD might be expected to result in cellular activation that would enhance extinction, but also potentiate reconsolidation. That this is seemingly not the case suggests the recruitment of specific cellular mechanisms with dissociable impacts on extinction and reconsolidation. One primary candidate for such a role is the calcineurin-nuclear factor kappa-light-chain-enhancer of activated B cells (NFkB) signaling pathway. In the hippocampus, NFkB inhibition both impaired fear memory reconsolidation and enhanced extinction (De La Fuente et al., 2011). Moreover, inhibition of calcineurin, which itself is a negative regulator of NFkB, impaired extinction and enhanced reconsolidation (De La Fuente et al., 2011, 2014). This pattern of results was partially replicated by calcineurin inhibition in the amygdala impairing cued fear memory extinction (Merlo et al., 2014). Therefore, if CB1R activation by CBD inhibits NFkB function, either directly (Curran et al., 2005) or via enhancement of calcineurin (Cannich et al., 2004), this would explain the behavioral reductions in fear expression, perhaps through downstream regulation of cytokine networks (Scholz et al., 2016).

## Summary

A growing body of literature provides compelling evidence that CBD has anxiolytic effects and recent studies have established a role for CBD in regulating learned fear by dampening its expression, disrupting its reconsolidation, and facilitating its extinction. The opposing effects of CBD on fear memory reconsolidation and extinction make it particularly attractive as a potential adjunct to psychological therapy as both may lead to lasting reductions in learned fear expression. Our novel data also suggests that CBD reduces the expression of fear memory related to both discrete and contextual cues. Although we found no effect of CBD on auditory fear extinction, decreasing fear expression during extinction without interfering in its encoding is still a useful property that has clinical implications. In this respect CBD might be an improvement over other available drugs used for treating the fear-related symptoms of phobias and PTSD, which can impair extinction (e.g., benzodiazepines) or have a less favorable side effect profile (e.g., antidepressants). As such, further research investigating the mechanisms underpinning CBD regulation of learned fear is warranted.

## Ethics statement

The study was conducted in accordance with ethical review by the Animal Welfare Ethical Review Board at the University of Nottingham and the Animals (Scientific Procedures) Act 1986, UK.

## Author contributions

RJ: Conducted the experiment investigating the effects of CBD on auditory fear memory expression and extinction; RJ, HD, and CS: Analyzed the data from this experiment. FG, JL, LB, and CS: Drafted the paper. All authors contributed to interpreting the original data, revising the draft and approving the final version of the paper.

### Conflict of interest statement

FG is co-inventor of the patent “Fluorinated CBD compounds, compositions and uses thereof. Pub. No.: WO/2014/108899. International Application No: PCT/IL2014/050023”; Def. US no. Reg. 62193296; 29/07/2015; INPI in 19/08/2015 (BR1120150164927). The other authors declare that the research was conducted in the absence of any commercial or financial relationships that could be construed as a potential conflict of interest.

## Abbreviations

AC: adenylyl cyclase
5-HT: serotonin
5-HT_1A_R: 5-HT_1A_ receptor
Δ9-THC: Δ9-tetrahydrocannabinol
BDNF: brain derived neurotrophic factor
BNST: bed nucleus of the stria terminalis
CB1R: cannabinoid receptor type 1
CBD: cannabidiol
ERK: extracellular signal-regulated kinase
i.c.v.: intracerebroventricular
IL: infralimbic
i.p.: intraperitoneal
NFkB: nuclear factor kappa-light-chain-enhancer of activated B cells
PKC: protein kinase C
PL: prelimbic
PTSD: post-traumatic stress disorder
SHR: spontaneously hypertensive rat
TrkB: tyrosine receptor kinase B.
