# Supplementary material for: Cannabidiol Regulation of Learned Fear: Implications for Treating Anxiety-Related Disorders
Source: Front Pharmacol. 2016 Nov 24;7:454. doi: 10.3389/fphar.2016.00454 (PMC5121237; doi:10.3389/fphar.2016.00454)
Supplement: Supplementary file 1 [file DataSheet1.DOCX]

Supplementary Material

**Cannabidiol regulation of learned fear: implications for treating anxiety-related disorders**

Regimantas Jurkus, Harriet LL Day, Francisco S Guimarães, Jonathan L Lee,

Leandro J Bertoglio,*****Carl W Stevenson

***Correspondence:** Carl W Stevenson, carl.stevenson@nottingham.ac.uk

**Supplementary Methods**

*Animals*

Male Lister hooded rats (280-340 g; Charles River, UK) were group housed on a 12 h light/dark cycle (lights on at 8:00) with free access to food and water. The study was conducted in accordance with ethical review by the Animal Welfare Ethical Review Board at the University of Nottingham and the Animals (Scientific Procedures) Act 1986, UK. All behavioral testing occurred during the animals’ light cycle.

*Drug preparation and administration*

Cannabidiol (STI Pharmaceuticals Ltd, UK) was suspended in 2% Tween 80 and 0.9% sterile saline on the day of use. Rats were injected with one of three doses of cannabidiol (5, 10, 20 mg/kg, i.p.) or vehicle (1 mL/kg, i.p.) 30 min before undergoing auditory fear extinction (see below). The doses of cannabidiol used were based on our previous studies examining its effects on fear memory processing, anxiety-like behaviour, and cardiovascular responsivity to stress [Guimarães et al. 1990; Resstel et al. 2009; Stern et al. 2012].

*Auditory fear conditioning and extinction*

The behavioral testing paradigm used (Figure 2A) was adapted from our previous studies [Stevenson et al. 2009; Fenton et al. 2014]. On Day 0, rats were habituated to two separate contexts (A and B; 10 min each). On Day 1, rats underwent tone habituation (five tones alone; 30 s, 80 dB, 4 kHz, 2 min inter-trial interval (ITI)) followed by auditory fear conditioning (five tones co-terminating with footshock; 0.5 s, 0.5 mA, 2 min ITI) in Context A. On Day 2, rats received a drug injection (see above) and 30 min later were subjected to extinction training (15 tones alone; 1 min ITI) in Context B. On Day 3, rats were tested for extinction recall (two tones alone, 1 min ITI) in Context B.

*Data Analysis*

Freezing, defined as the absence of movement except that related to respiration, served as the measure of learned fear. Freezing during tone-shock pairings on Day 1 and tone presentations on Days 2-3 was scored manually by two trained observers, one of whom was blind to drug treatment. Freezing was determined at 3 s intervals during tone presentations and the cumulative duration of freezing was calculated and expressed as a percentage of the 30 s tone. During extinction, the mean percentage of freezing during tones 1-5, 6-10, and 11-15 was calculated and used in the statistical analysis. During extinction recall, the mean percentage of freezing during both tones was calculated and used in the statistical analysis. Freezing during the 2 min period before tones were presented on Days 2 and 3 was calculated to infer contextual fear before extinction and extinction recall, respectively. Differences in freezing during fear conditioning between the groups to receive different drug treatments before extinction training the next day were analyzed using two-way analysis of variance (ANOVA), with dose and trial as between- and within- subject factors, respectively. Drug effects on tone-induced freezing during extinction were analyzed in the same way. The effects of drug given before extinction on tone-induced freezing during extinction recall the next day were analyzed using one-way ANOVA, with dose as the between subject factor. Drug effects on contextual fear before extinction and extinction recall were analyzed using two-way ANOVA, with dose and day as between- and within- subject factors, respectively. Freezing is presented as the mean + SEM. Post-hoc comparisons were conducted using the Tukey’s test. The level of significance for all comparisons was set at P<0.05.

**Supplementary Results**

Freezing during fear conditioning is shown in Figure 2B. Two-way ANOVA revealed no main effect of group (F_(3,44)_=0.22, P>0.05) or group x trial interaction (F_(12,176)_=0.42, P>0.05). This indicates that there were no differences in fear conditioning between the rats to receive vehicle (n=11), 5 mg/kg (n=10), 10 mg/kg (n=11), or 20 mg/kg (n=11) of cannabidiol before extinction the following day. The effects of cannabidiol on freezing in response to tone presentations during extinction is shown in Figure 2C. Two-way ANOVA revealed a significant dose x trial interaction (F_(6,78)_=3.99, P<0.01). Post-hoc analysis revealed that 20 mg/kg of cannabidiol significantly decreased freezing during the first tone block (tones 1-5), compared to 5 mg/kg and vehicle (P<0.05). This indicates that the highest dose of cannabidiol reduced auditory fear expression. Freezing in response to tone presentations during extinction recall is shown in Figure 2D. One-way ANOVA revealed no dose effect (F_(3,39)_=0.55, P>0.05), indicating that there were no effects of cannabidiol on extinction memory encoding. Freezing before tone presentations during extinction and extinction recall is shown in Figures 2E and 2F, respectively. Two-way ANOVA revealed a significant dose x day interaction (F_(3,39)_=2.92, P<0.05). Post-hoc analysis revealed that, compared to vehicle, freezing before tone presentations during extinction was significantly decreased with each dose of cannabidiol (P<0.01). In contrast, there were no differences between the groups in freezing before tone presentations during extinction recall. This indicates that, while contextual fear expression before extinction was reduced acutely by cannabidiol, there were no lasting drug effects on contextual fear before extinction recall testing the next day.

**Supplementary References**

Guimarães FS, Chiaretti TM, Graeff FG, Zuardi AW (1990) Antianxiety effect of cannabidiol in the elevated plus-maze. *Psychopharmacology (Berl)* **100**:558-559.

Fenton GE, Pollard AK, Halliday DM, Mason R, Bredy TW, Stevenson CW (2014) Persistent prelimbic cortex activity contributes to enhanced learned fear expression in females. *Learn Mem* **21**:55-60.

Resstel LB, Tavares RF, Lisboa SF, Joca SR, Corrêa FM, Guimarães FS (2009) 5-HT1A receptors are involved in the cannabidiol-induced attenuation of behavioural and cardiovascular responses to acute restraint stress in rats. *Br J Pharmacol* **156**:181-188.

Stern CA, Gazarini L, Takahashi RN, Guimarães FS, Bertoglio LJ (2012) On disruption of fear memory by reconsolidation blockade: evidence from cannabidiol treatment. *Neuropsychopharmacology* **37**:2132-2142.

Stevenson CW, Meredith JP, Spicer CH, Mason R, Marsden CA (2009) [Early life programming of innate fear and fear learning in adult female rats.](http://www.ncbi.nlm.nih.gov/pubmed/18996416) *Behav Brain Res* **198**:51-57.
